# *Bacillus subtilis* Bactofilins Are Essential for Flagellar Hook- and Filament Assembly and Dynamically Localize into Structures of Less than 100 nm Diameter underneath the Cell Membrane

**DOI:** 10.1371/journal.pone.0141546

**Published:** 2015-10-30

**Authors:** Jihad El Andari, Florian Altegoer, Gert Bange, Peter L. Graumann

**Affiliations:** 1 SYNMIKRO, LOEWE Center for Synthetic Microbiology, and Department of Chemistry, Philipps Universität Marburg, Marburg, Germany; 2 University of Freiburg, Faculty of Biology, Schaenzlestrasse 1, D–79104, Freiburg, Germany; University of Groningen, Groningen Institute for Biomolecular Sciences and Biotechnology, NETHERLANDS

## Abstract

Bactofilins are a widely conserved protein family implicated in cell shape maintenance and in bacterial motility. We show that the bactofilins BacE and BacF from *Bacillus subtilis* are essential for motility. The proteins are required for the establishment of flagellar hook- and filament structures, but apparently not for the formation of basal bodies. Functional YFP fusions to BacE and to BacF localize as discrete assemblies at the *B*. *subtilis* cell membrane, and have a diameter of 60 to 70 nm. BacF assemblies are relatively static, and partially colocalize with flagellar basal bodies, while BacE assemblies are fewer per cell than those of BacF and are highly mobile. Tracking of BacE foci showed that the assemblies arrest at a single point for a few hundred milliseconds, showing that a putative interaction with flagellar structures would be transient and fast. When overexpressed or expressed in a heterologous cell system, bactofilins can form filamentous structures, and also form multimers as purified proteins. Our data reveal a propensity for bactofilins to form filaments, however, in *B*. *subtilis cells*, bactofilins assemble into defined size assemblies that show a dynamic localization pattern and play a role in flagellar assembly.

## Introduction

Bactofilins are a newly identified class of proteins sharing the bactofilin (DUF583) domain and are widespread in most bacterial species analyzed so far [1, 2]. Many of the genomes containing bactofilin genes have multiple paralogous copies, which can be organized in an operon-like structure, or can be present at distinct genomic sites. In *Caulobacter crescentus* and in *Myxococcus xanthus*, bactofilins recruit other proteins to specific subcellular sites: a cell wall synthetic enzyme to the tip of the stalk structure [1], or a GTPase regulating motility to the cell poles, respectively [3]. Thus, bactofilins have been implicated as scaffolding proteins in bacteria.

Bactofilins have been shown to form stable filamentous structures *in vitro*, even in the absence of any nucleotide cofactor [1]. BacM purified from *M*. *xanthus* also forms filaments when purified directly from *M*. *xanthus* cells [4]. Therefore, this conserved class of proteins has been proposed to assemble at distinct subcellular sites, serving as multimeric scaffold for the assembly of other proteins. It has recently been shown that the domain of unknown function (DUF) 583 can act as a central polymerizing module in bactofilins, and adopts a beta-helical structure [5]. The domain is flanked by terminal segments that are predicted to be disordered, and are often highly charged. A majority of bactofilin homologues are supposedly soluble, except for some enterobacterial orthologs that contain an N-terminal transmembrane region, with the DUF583 domain being located in the bacterial cytoplasm. Overproduction of BacA and BacB in *Caulobacter crescentus* leads to the formation of visibly extended curved filaments [1]. Although BacA and BacB are predicted to be soluble, they co-localize at the neck of the stalk, and indeed co-sediment with the membrane. Localization at the stalked pole occurs during the swarmer-to-stalked-cell transition, and thus at a defined time point in the cell cycle of *C*. *crescentus*. The two proteins co-localize with PbpC (Penicillin-binding protein C), which is a peptidoglycan synthetase. This suggests that BacA and BacB play a role in the stalk biogenesis, and indeed, a *bacA* deletion resulted in a decrease in stalk length, approximately to 45% compared to the wild type [1]. Upon the deletion of *M*. *xanthus* bactofilin genes encoding for BacN, BacO, or BacP, cell morphology was found to be normal, while a strain lacking BacP had impaired social motility [1]. Interestingly, in *Shewanella oneidensis*, a fluorescent band was observed at the cell division site for bactofilin SO1162-*mCherry*, indicating a possible role in cytokinesis [1]. There is evidence suggesting that BacM plays a role for cell morphology in *M*. *xanthus* [4]. The bactofilin was shown to be involved in tolerance against cell wall-targeting antibiotics in *M*. *xanthus* and to assemble into filamentous structures *in vivo*, which were shown to be bundles of fibres *in vitro* [4]. Another putative bactofilin is HPG27_1480, also known as *Helicobacter pylori ccmA*, a homolog of *Proteus mirabilis ccmA*. Interestingly, the deletion of *H*. *pylori ccmA* resulted in a transition from curved to straight rod morphology [6], suggesting that it is involved in cell shape maintenance. Recently, it was shown that *M*. *xanthus* BacP plays an important role in type IV pili (T4P)-dependent motility [3], also referred as S-motility. T4P assembly is associated to cell polarity, which is temporally and spatially regulated through a series of assembly and disassembly events of the apparatus, between cell poles, in a way that determines the new leading pole and thereby directionality. BacP interacts directly with SofG (small GTPase) and as a result localizes both PilB and PilT (two motor ATPases) to the same pole followed by the intervention of MglA (Ras-like GTPase) that eventually sorts both proteins to opposite cell poles [3]. It is not yet clear how the widely conserved class of proteins performs its functions in motility and cell shape maintenance at a molecular level.

Paralogous bactofilin genes *yhbE* and *yhbF* from *Bacillus subtilis*, whose products we term BacE and BacF, are highly conserved in most *Bacillus* species. *B*. *subtilis yhbE yhbF* double mutants showed reduced motility, and in a yeast two hybrid screen, *yhbE* and *yhbF* paralogs from *Treponema pallidum* were found to interact with flagellar proteins FliY and FliS [7].

The assembly of the flagellum, which consists of the three major parts, engine (basal body), propeller (filament) and universal joint (hook), is a highly coordinated process [8], driven by the proton motive force [9, 10]. The basal body assembles at the cell membrane as a ring structure that extends well into the cytosol, whereas the hook, filament—and in Gram negative bacteria components of the machinery that span the outer membrane—need to be exported via the flagellar type III secretion system (fT3SS), which assembles in the center of the basal body. FliY is a protein in the basal body complex of the flagellum [11, 12]. It is a component of the C-ring, and connects the chemotactic input to the rotational state of the flagellum [13]. FliY belongs to the so-called “switch complex”, which also contains proteins FliG, FliM, and in some species additional proteins [14, 15]. FliY contributes to chemotactic regulation via its conserved CheC-like phosphatase domain that dephosphorylates CheY [16, 17]. FliS, on the other hand, is a chaperone of flagellin and prevents futile polymerization of its client in the cytosol [18–20]. The flagellin-FliS complex is then recognized by the cytosolic domain of FlhA, a core part of the fT3SS prior to secretion [21–23]. It has been unclear what relevance the interaction between spirochaete bactofilins and FliY and FliS may have.

We show that *B*. *subtilis* bactofilins play a defined role in the assembly of the filament as well as of the hook in the *Bacillus* flagellum. Thus, bactofilins are important for a step mediating the export of hook and flagellin through the secretion system. However, bactofilin fusions to YFP show only partial colocalization with flagellar assemblies, or highly dynamic movement in case of BacE, revealing that interactions with flagellar proteins are transient, yet essential for the full maturation of the flagellum. Our data reveal a novel dynamic mode of action of bactofilins and place them into a molecular framework of flagellar assembly.

## Material and Methods

### Strains and growth conditions

All *Bacillus subtilis* strains were grown in Luria—Bertani (LB) broth or on LB plates at 30°C. *Escherichia coli* strain XL1-Blue (Stratagene) was used for the construction and propagation of plasmids and was grown on LB plates and in LB broth at 37°C. Antibiotics were added, when necessary, at the following concentrations: 100 μg ml^-1^ Ampicillin (Amp), 10 μg ml^-1^ kanamycin (Kan), 10 μg ml^-1^ tetracycline (Tet), 100 μg ml^-1^ spectinomycin (Spec), 5 μg ml^-1^ chloramphenicol (Cm), and 1 μg ml^-1^ erythromycin plus 25 μg ml^-1^ lincomycin (Mls). For fluorescence microscopy, cells were grown at 30°C in S7_50_ minimal medium [24].

Soft agar plates were freshly prepared for swimming motility assays using 25 ml of LB fortified with 0.3% Bacto agar, dried in laminar flow hood prior to inoculation. Plates were centrally inoculated by a sterile tip from individual colonies and plates were analyzed after 18 h incubation at 37°C.

### Strain constructions

All constructs were eventually introduced into wild type strain PY79 by natural competence. Moreover, transformation with genomic DNA of previously constructed *Bacillus subtilis* strains (PY79 and 3610) was also used in this study. All *Bacillus* strains used are listed in Table 1, *E*. *coli* strains in S1 Table and primers in S2 Table.

**Table 1:** *Bacillus subtilis* strains used in this study.

**3610**	wild type	undomesticated
RL2664	*hag*::*mls*	[37]
DS7673	*amyE*::*P*_*D-3*_ *P*_*fla-che*_ *-flgE* ^*T123C*^ *cat*	[25]
DS4668	*WDflgE mls*	[25]
DS1916	*amyE*::*P*_*hag*_ *-hag* ^*T209C*^	[30]
**PY79**	wild type (laboratory strain)	[38]
DS313	*sigD*::*tet*	[31]
JEA100	*yhbEF*::*tet*	this study
JEA101	*yhbE-yfp cat*	this study
JEA102	*P*_*xyl*_ *-yfp-yhbE cat*	this study
JEA103	*yhbF-yfp cat*	this study
JEA104	*P*_*xyl*_ *-yfp-yhbF cat*	this study
JEA105	*P*_*xyl*_ *-yfp-fliG cat*	this study
JEA106	*yhbEF*::*tet P*_*xyl*_ *-yfp-fliG cat*	this study
JEA107	*amyE*::*P*_*fla-che*_ *-fliM-cfp spec*	this study
JEA108	*yhbEF*::*tet amyE*::*P*_*fla-che*_ *-fliM-cfp spec*	this study
JEA109	*P*_*xyl*_ *-yfp-yhbF cat amyE*::*P*_*fla-che*_ *-fliM-cfp spec*	this study
JEA110	*amyE*::*P*_*xyl*_ *-yfp-yhbE spec*	this study
JEA111	*yhbEF*::*tet amyE*::*P*_*xyl*_ *-yhbEF spec*	this study
JEA112	*yhbEF*::*tet amyE*::*P*_*xyl*_ *-yfp-yhbE spec*	this study
JEA113	*amyE*::*P*_*xyl*_ *-yfp-yhbF spec*	this study
JEA114	*yhbEF*::*tet amyE*::*P*_*xyl*_ *-yfp-yhbF spec*	this study
JEA115	*amyE*::*P*_*xyl*_ *-yn-yhbE spec*	this study
JEA121	*hag*::*mls*	this study
JEA124	*hag*::*mls amyE*::*P*_*hag*_ *-hag* ^*T209C*^	this study
JEA125	*yhbEF*::*tet hag*::*mls amyE*::*P*_*hag*_ *-hag* ^*T209C*^	this study
JEA126	*ΔflgE*	this study
JEA127	*ΔflgE amyE*::*P*_*D-3*_ *P*_*fla-che*_ *-flgE* ^*T123C*^	this study
JEA128	*yhbEF*::*tet ΔflgE amyE*::*P*_*D-3*_ *P*_*fla-che*_ *-flgE* ^*T123C*^	this study
JEA129	*hag*::*mls ΔflgE amyE*::*P*_*D-3*_ *P*_*fla-che*_ *-flgE* ^*T123C*^	this study
JEA130	*amyE*::*P*_*xyl*_ *-yhbE-YFP*	this study
JEA131	*amyE*::*P*_*xyl*_ *-yhbF-YFP*	this study
JEA132	*P*_*xyl*_ *-yfp-fliY cat*	this study
JEA133	*yhbEF*::*tet P*_*xyl*_ *-yfp-fliY cat*	this study
JEA134	*yhbEF*::*tet P*_*xyl*_ *-yhbE*	this study
JEA135	*yhbEF*::*tet P*_*xyl*_ *-yhbF*	this study

To generate *yhbEF*::*tet* (*ΔbacEF*), the region upstream of *yhbE* and downstream of *yhbF* was PCR-amplified using the primer pair 2558/2559 and digested with *Apa*I and *Eco*RI. The fragment was then ligated into pSG1729. The construct was digested with *Hin*dIII and ligated with the *tet* cassette, which had been amplified from pDG1515 using 4168/4169. The deletion cassette was amplified from the final construct and introduced into PY79 and was confirmed by comparing the sizes of the amplicons (wt versus *yhbEF*::*tet*) using 2558/2559 and followed by sequencing. DNA of this strain was used as well to generate a deletion background with cysteine-labeled hook and flagella, and for other strains (Table 1). The complementation construct was generated by amplifying the coding sequence of *yhbEF* using primers 2215/4341, followed by digestion with *Apa*I and *Eco*RI. This fragment was introduced into pSG1193 that integrates to *amyE* locus. For complementation with each bactofilin gene individually, the coding sequence of each *yhbE* and *yhbF* was amplified using 2215/5203 and 2238/4341, respectively. The DNA fragments were digested with *Apa*I and *Eco*RI and were cloned into pSG1193. All downward primers used for genetic complementation contain a stop codon that arrests translation before the *yfp* gene in the plasmid.

Deletion strain *hag*::*mls* (*Δhag*) was made using DNA from strain RL2664, which was complemented by introducing DNA of (DS1916) *amyE*::*P*_*hag-*_ *hagT* ^*209C*^.

The in-frame (markerless) deletion of *ΔflgE* was made by using DNA of DS4668, which contains pDP306 with Mls resistance that was integrated by single-crossover. The plasmid was then evicted by performing a series of dilutions and outgrowths in LB containing no Mls at 22°C as described by [25]. The mutant was complemented with DS7673 to create *amyE*::*P*_*D-3*_ *P*_*fla-che*_ *-flgE* ^*T123C*^ *cat*. The generated constructs (*hag*::*mls*::*amyE*::*P*_*hag-*_ *hag* ^*T209C*^ *and flgE*::*amyE*::*P*_*D-3*_ *P*_*fla-che*_ *-flgE* ^*T123C*^) were also studied in a bactofilin deletion background by DNA transformation of *yhbEF*::*tet* strain.

BacE-YFP and BacF-YFP were created by amplifying 500 bp of the 3’ end of *yhbE* and *yhbF* using the following primers 2213/2214 and 2240/2241 respectively. The amplicons were digested using *Apa*I and *Eco*RI and ligated to pSG1164. Moreover, YFP-BacE, YFP-BacF, YFP-FliG, and YFP-FliY were constructed by amplifying 500 bp of the 5’ end of the corresponding genes using the following primers 2215/2216, 2238/2239, 2926/2927, and 4922/4923 respectively. Amplified DNA was digested using *Apa*I and *Eco*RI and was ligated to pHJDSI. All the above-mentioned fusions replaced the native genes at the corresponding original locus. The pHJDSI fluorescent fusions are expressed under the control of a xylose inducible promoter, whereas pSG1164 fusions are driven by their original corresponding gene promoters. To express YFP-BacE or BacE-YFP ectopically from the *amyE* locus with a xylose inducible promoter, the entire coding sequence of *yhbE* was amplified with 2215/2214, digested with *Apa*I and *Eco*RI and subsequently ligated to pSG1729 or to pSG1193, respectively. YFP-BacF and BacF-YFP were also cloned at the *amyE* locus. The full-length gene was amplified using 2238/2241 and was digested and ligated as described for BacE. FliM-CFP fusion was reintroduced into PY79 as described in [26].

### Microscopy

Fluorescence microscopy was performed using a Zeiss Axio Imager A1 equipped with an EVOLVE EMCCD camera (Photometrics) and a TIRF objective with an aperture of 1.45. Picture acquisition was done using VisiView (2.1.2). Time-lapse microscopy was performed using a 515 nm laser illumination. Super resolution microscopy was done using Leica G-STED SP8 microscope. Images were acquired with three or four line scans at 200 Hz. Further analysis was done with Metamorph 6.5 (MDS Analytical Technologies) and ImageJ (National Institutes of Health, Bethesda, MD), MTrackJ (ImageJ plugin) was used to generate the tracks.

For fluorescent microscopy of the cysteine-labeled hook and flagella, cells were pelleted (0.5 ml of LB broth at 0.5–2.0 OD_600_) by delicate centrifugation at 3500–5000 rpm for 10 min to avoid the shearing of the flagella or hook, and washed once with 1 ml T-Base buffer [15 mM (NH_4_)_2_SO_4_, 80 mM K_2_HPO_4_, 44 mM KH_2_PO_4_, 3.4 mM sodium citrate, and 3.0 mM MgSO_4_·6H_2_0]. Cells were resuspended in 50 μl of T-Base buffer containing 5 μg/ml Alexa Fluor 488 C_5_ maleimide (Molecular Probes), and incubated for 5 min at room temperature. Cells were then washed once with 500 μl T-Base buffer.

### Protein expression and purification

Full-length *yhbE* and *yhbF* were amplified using the following primers: 4309/4305 or 4310/4305 for *his*_*6-*_ *yhbE/yhbE* and 4306/4308 or 4307/4308 for *his*_*6-*_ *yhbF*/*yhbF*, and were each cloned into pET16b (Novagen) or pET24d (Novagen), after digestion with *Nco*I and *Bam*HI. Each construct was expressed individually or was coexpressed with the second protein in *Escherichia coli* BL21 (DE3). Purification was done using Ni-ion affinity and size exclusion chromatography. Cells were grown in 1 l LB broth medium at 37°C in a shaking incubator at 200 rpm and induced for protein expression with 1 mM IPTG at approximately an OD_600_ of 0.6. Cells were then transferred into 30°C shaking incubator and incubated for 2.5 h followed by harvesting and storing at – 80°C. The pellet was resuspended in 10 ml lysis buffer per gram of cells containing 20 mM Na-HEPES pH 7.5, 250 mM NaCl, 10 mM MgCl_2_, 10 mM KCl, and 1 tablet of Mini EDTA-free, EASYpack (Roche) protease inhibitor cocktail and pressed through M1-10L Microfluidizer (Microfluidics). The lysate was then centrifuged for 40 min at 16,000 rpm using JA-25.50 rotor (Beckman Coulter). The supernatant was applied to 1 ml HisTrap HP column (GE Healthcare) that was previously equilibrated with 5 column volumes of lysis buffer containing 40 mM imidazole pH 7.5 and eventually eluted with lysis buffer 500 mM imidazole pH 7.5. Proteins were concentrated, using Amicon Ultracel-10 K (Millipore), to 30 mg^-1^ approximately. Subsequently, proteins were separated by size-exclusion chromatography using an S200/26–60 column (GE Healthcare) in buffer A containing 20 mM HEPES-Na, pH 7.5, 250 mM NaCl, 10 mM KCl, 10 mM MgCl_2_. The absorption profile of protein samples at 280 nm was measured and protein fractions from different absorption maxima were analyzed by SDS-PAGE. Protein-containing fractions were pooled and concentrated to 30 mg ml^−1^.

### Leifson staining

To visualize flagellar filaments of bacteria, Leifson staining was used [27]. Solution A (256.8 mM NaCl) and Solution B (17.6 mM tannic acid) were prepared in dH_2_O and Solution C (259.1 mM pararosaniline acetate and 92.64 mM pararosaniline hydrochloride) in 95% EtOH. Solution A and B were mixed (1:1 ratio) and the mixture was added to solution C (2:1 ratio). Cells were harvested and washed as described for fluorescence microscopy of flagella and hook. Cells (10–15 μl) were added to the slide and were air-dried. The stain was added and incubated at room temperature for 5–7 min, until a golden film was developed on the surface of the dye. The dye was washed gently with water and the slide was air-dried.

### Schneider cell culture and transient transfection

*D*. *melanogaster* S2 Schneider cells [28] were grown in Schneider's Drosophila medium (Lonza Group Ltd.) supplemented with 5–10% (v/v) fetal calf serum (FCS) at 25°C and cells were transferred every 48 h to maintain optimal growth conditions. Prior to transfection, S2 cells were spread in a 6–well plate at 1×10^6^ cells per well in 3 ml medium containing 5% FCS for 18 h at 25°C. Vectors (0.3 μg) were mixed with 10 μl of X-tremeGENE HP DNA Transfection Reagent (Roche) in 200 μl serum-free medium. The mixture was incubated at room temperature for 15 min and the volume was made up to 1 ml with serum-free medium. Prior to adding the mixture, the growth medium was removed and cells were washed 1× with serum-free medium. After adding the transfection reaction, cells were incubated for 18 h incubation. The mixture was then removed and replaced by 3 ml of growth medium containing 10% FCS. CuSO4 to a final concentration of 1 mM was used to induce the production of proteins and cells were analyzed after 18 h.

To express YhbE-YFP and YhbF-YFP, full-length coding sequences of *yhbE* and of *yhbF* were separately amplified using primers 2215/2573 and 2238/2574, respectively. Each fragment was digested with *Apa*I and *Eco*RV and ligated to pFD1 (S1 Table). To express YFP-YhbE and YFP-YhbF, pOTJ1 was constructed by inserting a fragment containing the RBS, YFP and MCS of pSG1729 (S1 Table) that had been digested with *Xba*I and *Eco*RV.

### Western blotting

*B*. *subtilis* cultures (1 ml) were harvested by centrifugation. The pellet was resuspended in lysis buffer (20 mM Tris-HCl [pH 7.0], 10 mM EDTA, 1 mg ml^-1^ lysozyme, 10 g ml^-1^ DNase I, 100 g ml^-1^ RNase I, 1 tablet of Mini EDTA-free, EASYpack (Roche) protease inhibitor cocktail), and incubated for 30 min at 37°C. Proteins were separated by running 12% sodium dodecyl sulfate-polyacrylamide gel electrophoresis (SDS-PAGE) and were transferred onto nitrocellulose membrane followed by blocking with 5% milk in PBST (80 mM Na_2_HPO_4_, 20 mM NaH_2_PO_4_, 100 mM NaCl, 0.2% (v/v) Tween-20). Proteins were probed using a 1:500 dilution (rabbit-α-GFP) or a 1:8000 dilution of anti-flagellin antiserum, and secondary antibody was added (goat-α-rabbit-antibody in 1:10000 dilution) after a series of washing steps with PBST. Solution A (100 mM Tris pH 8.5, 2.5 mM Luminol, and 0.4 mM Coumaric acid) and Solution B (100 mM Tris pH 8.5, 0.02% (v/v) H_2_O_2_) were prepared and mixed followed by incubation for 2 min for chemoluminescence detection with ChemiDocTM MP System (BIO-RAD). Intensity of bands was quantified using ImageJ (NIH) as the specified lanes were plotted and the corresponding peak area was measured in arbitrary units, which was eventually indicated as percentages.

## Results

### BacE and BacF are essential for motility in *Bacillus subtilis*

**Fig 1:**
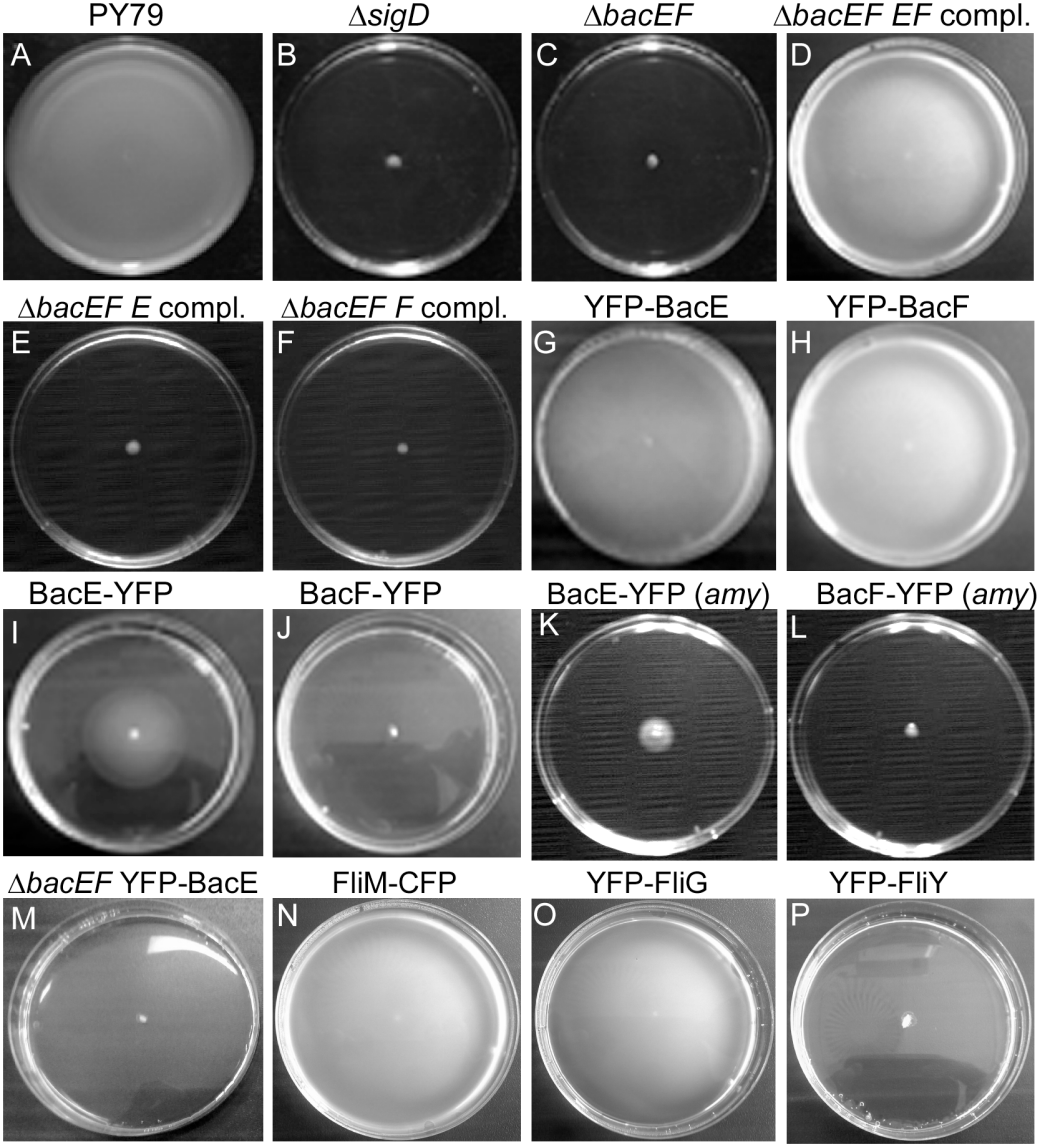
Motility assays of *Bacillus subtilis* wild type and mutant strains. Inoculums are placed in the center of 0.3% agar plates, and spreading is analyzed after 18 hours of incubation. A) wild type, B) Δ*sigD* = sigma D deletion (DS313), C) Δ*bacEF* = deletion of bactofilin genes (JEA100), D) Δ*bacEF EF* compl = deletion of bactofilin genes, expression of *bacE* and *bacF* from the amylase locus (JEA111), E) deletion of bactofilin genes, expression of *bacE* from the amylase locus (JEA134), F) deletion of bactofilin genes, expression of *bacF* from the amylase locus (JEA135), G) YFP-BacE (JEA102), and H) YFP-BacF (JEA104), I) BacE-YFP (JEA101), J) BacF-YFP (JEA103), K) expression of *bacE-yfp* from the amylase locus in wild type cells (JEA130), L) expression of *bacF-yfp* from the amylase locus in wild type cells (JEA131), M) expression of YFP-BacE from the amylase locus in the *bacEF* deletion strain (JEA112), N) FliM-CFP (JEA107), O) YFP-FliG (JEA105), P) YFP-FliY (JEA132).

Based on the presence of a DUF583 domain, two genes, *yhbE* and *yhbF*, can be identified to encode the bactofilins BacE and BacF, respectively, in *Bacillus subtilis*. Both genes are in a putative operon together with the upstream *yhbD* open reading frame, which is only conserved in Bacilli and in the closely related Clostridia [29], whereas bactofilins genes are found in many bacteria. BacE and BacF show 32% sequence identity to each other and 45% and 39% sequence identity to e.g. their *Bacillus cereus* orthologs. The insertion of a phleomycin cassette into the *yhbEF* locus was reported to result in a severe reduction in swimming motility in *B*. *subtilis* strain 168 [7]. To address the functional roles of BacE and BacF in *B*. *subtilis* strain PY79, we created a gene deletion of the *yhbEF* locus, by exchanging the *yhbE* and *yhbF* genes for a tetracycline resistance cassette. In contrast to wild type cells (Fig 1A), double mutant cells showed a complete loss of swimming motility (Fig 1C, swimming is assayed on soft agar whose surface contains enough water to support flagellar-based swimming), similar to the loss of sigma D, the master regulator for late motility genes (Fig 1B). There was no detectable growth defect associated with the double deletion. When both *yhbE* and *yhbF* were integrated into the amylase locus on the chromosome, under the control of the inducible xylose promoter, cells regained the ability to swim after addition of xylose (Fig 1D). This verifies that the two proteins are essential for swimming motility in *B*. *subtilis*. In spite of various attempts, we did not succeed in deleting the individual genes, for reasons that are unclear to us. To test if both, or only one of the genes are involved in motility, we integrated *bacE* or *bacF* at the amylase locus in the *bacEF* deletion strain. Neither expression of BacE or of BacF alone rescued motility (Fig 1E and 1F), therefore, both bactofilin genes need to be present and expressed in cells in order to obtain full motility in *B*. *subtilis*.

We generated fluorescent protein fusions to localize the gene products. C-terminal fusion proteins for bactofilins (and for other proteins in this work) within the original gene locus were constructed such that downstream genes (e.g. *bacF* in the *bacE-yfp* strain) were driven by the xylose-inducible promoter. In the presence of xylose for BacE-YFP, C-terminal fusions to either protein resulted in the generation of partially motile (BacE-YFP) or of non-motile (BacF-YFP) cells (Fig 1I and 1J), showing that even a modification of BacE or of BacF at their C-terminus results in a motility defect. Interestingly, the expression of BacE-YFP or of BacF-YFP from an ectopic site on the chromosome also compromised motility (Fig 1K and 1L), revealing that the C-terminal fusions had a dominant negative effect, possibly based on defective multimerization.

**Fig 2:**
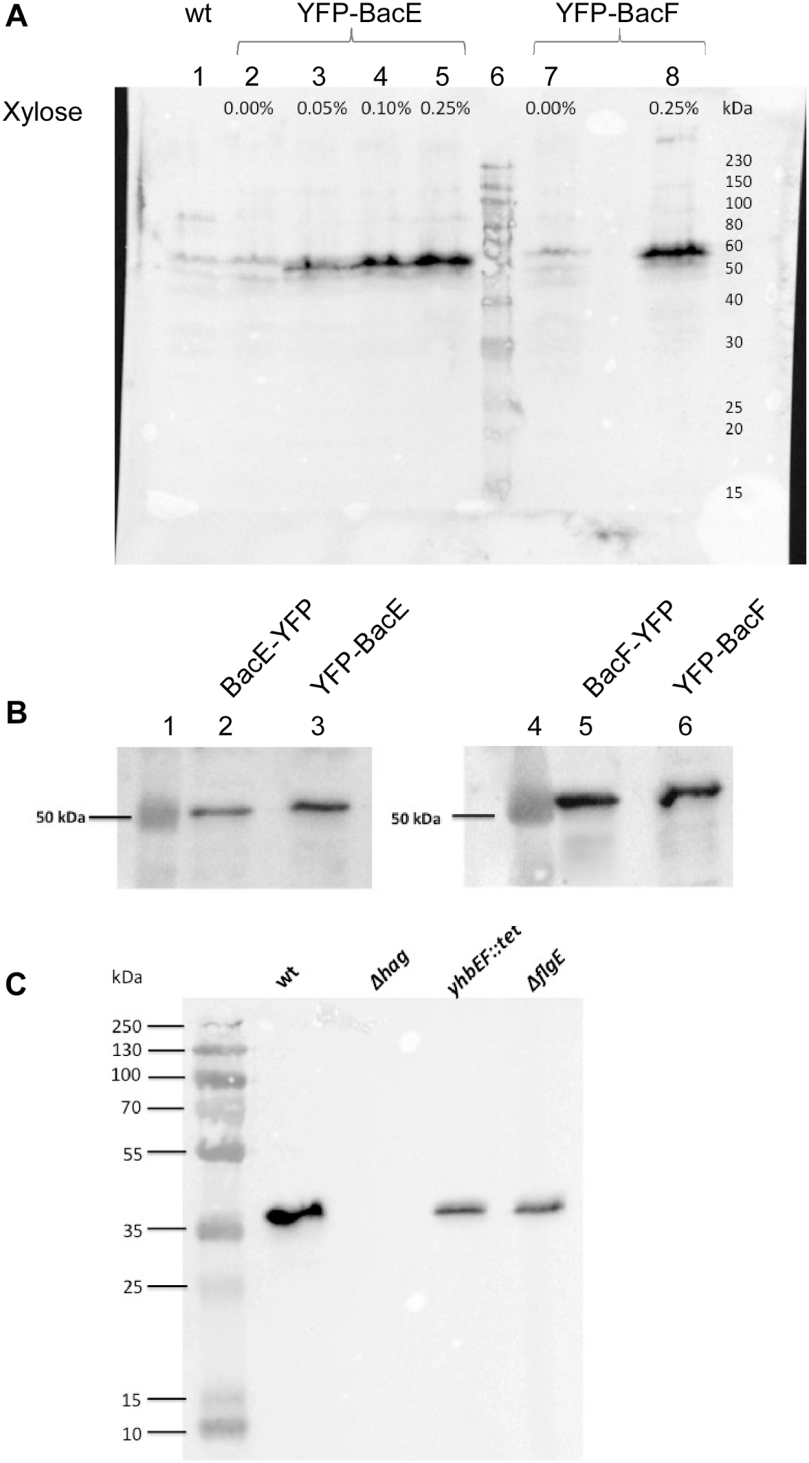
Western blotting of bactofilin fusion proteins, using anti-GFP antiserum. All lanes are normalized to optical cell density. A) Expression levels of N-terminal fusions of BacE (JEA102) or of BacF (JEA104), driven by different concentration of the inducer xylose; B) expression of BacE-YFP (JEA101) and of BacF-YFP (JEA103) driven by the original promoter at the original gene locus, compared to induction of YFP-BacE (JEA102) and YFP-BacF (JEA104) with 0.1% xylose. C) Western blot analysis using anti flagellin antiserum. “wt” = PY79 wild type cells, “Δ*hag*” = flagellin mutant cells (JEA121), “*yhbEF*::*tet*” = bactofilin mutant cells (JEA100), “Δ*flgE*” = hook-mutant cells (JEA126). Note that in wild type cells, flagellin from flagella is also present to some extent in the cell extract, in addition to internal flagellin, while there are no external flagella in bactofilin and hook mutant cells.

N-terminal fluorescent protein fusions generated in this work were either integrated at the *amy* site, such that the fusion is expressed as a merodiploid version driven by the xylose promoter, or at the original gene locus, such that the fusion and all downstream genes are induced by the xylose promoter, ruling out polar effects. We generated YFP fusions to the N-termini of either BacE or BacF, integrated at the original gene locus, such that the fusions were expressed as sole source of the proteins in the cells. The fusions were expressed as full-length proteins (Fig 2A) and at physiological levels (Fig 2B), and fully supported motility (Fig 1G and 1H), showing that these fusions functionally replace the wild type bactofilins. As expected, when YFP-BacE was expressed from the *amy* site in the bactofilin double mutant background (from here on simply termed “bactofilin mutant”), the single functional fusion did not restore spreading on 0.3% agar surfaces (Fig 1M). These data show that the generation of single non-functional alleles of *bacE* or *bacF* interfere with motility, in agreement with the finding that the presence of both proteins is required for motility, and show that even the expression of a non-functional protein interferes with cell motion in wild type cells.

### BacE and BacF are essential for flagellar hook- and filament assembly, but apparently not for the establishment of basal bodies

**Fig 3:**
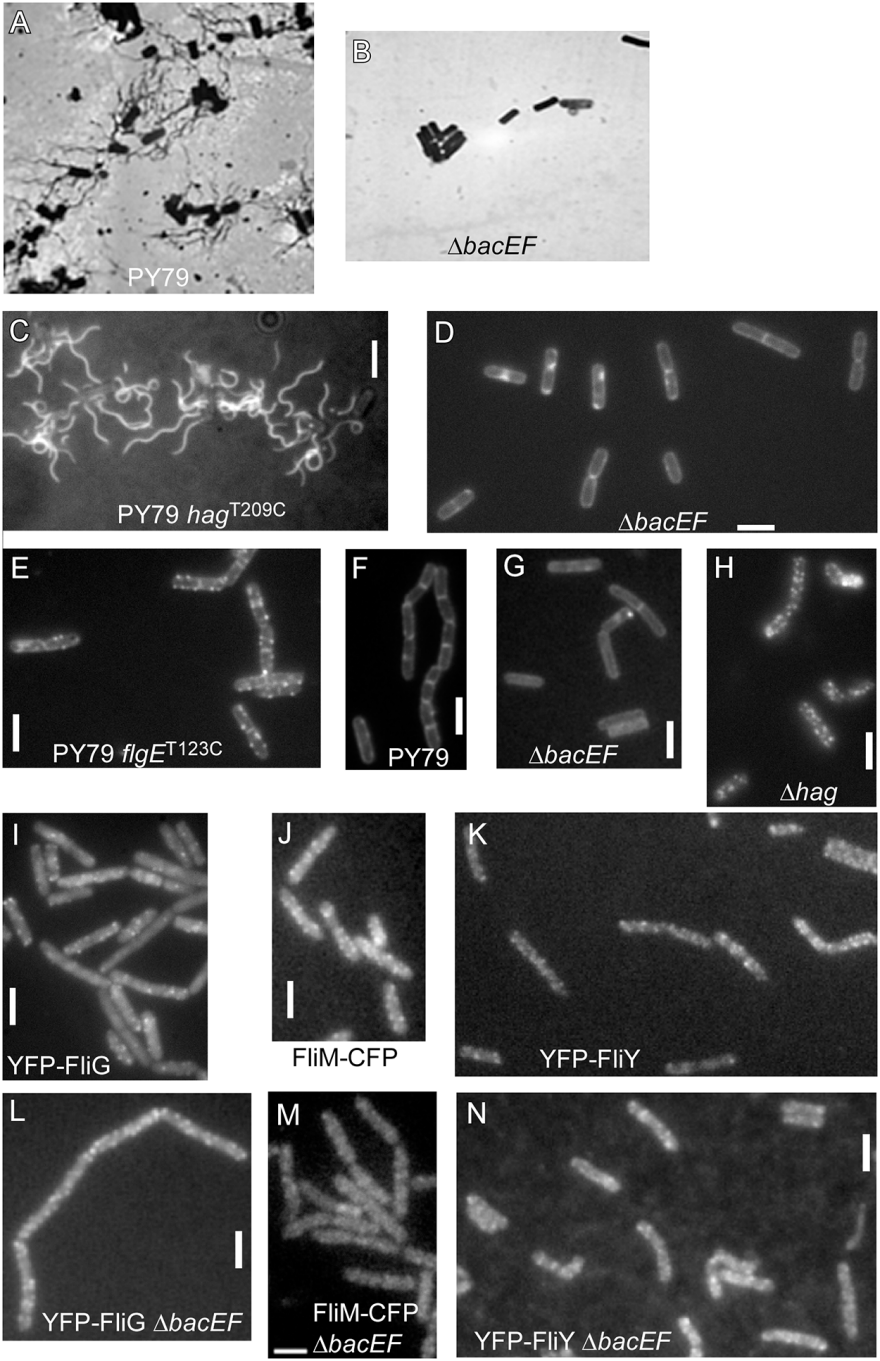
Staining of different components of the *B*. *subtilis* flagellum in cells during late exponential growth. A-B) Leifson staining of flagella, bright field images, A) wild type cells, B) *bacEF* mutant cells, C-D) Fluorescent staining of filaments, A) PY79 wild type cells expressing a variant of flagellin carrying an engineered cysteine, (JEA124), B) bactofilin double mutant cells expressing the cysteine-carrying variant of flagellin (JEA125). E-H) fluorescent staining of hooks, E) wild type cells expressing the cysteine-carrying FlgE^T123C^ protein (JEA127), F) control cells without the cysteine allele, G) Δ*bacEF* double mutant cells expressing the cysteine-carrying hook protein (JEA128), H) flagellin (gene is called *hag*) mutant cells expressing the cysteine-carrying hook protein (JEA129). I) cells expressing YFP-FliG (JEA105), J) cells expressing FliM-CFP (JEA107), K) cell expressing YFP-FliY (JEA132), L) bactofilin double mutant cells expressing YFP-FliG (JEA106), M) bactofilin double mutant cells expressing FliM-CFP (JEA108), N) bactofilin double mutant cells expressing YFP-FliY (JEA133). White bars 2 μm.

Visual inspection of bactofilin mutant cells revealed a complete absence of movement under the microscope. To test if this correlates with a defect in the structure of the flagellum, we performed Leifson flagellar staining, which visualizes flagellation of PY79 (Fig 3A). In agreement with their inability to be motile, bactofilin mutant cells were completely devoid of flagella indicated by the absence of stained flagellar filaments (Fig 3B). To confirm this observation, we employed a strain containing a *hag* allele that encodes a flagellin with an engineered surface cysteine [30]. This strain allows staining of the filament by a cysteine-reactive fluorescent dye added to the cells. Fluorescence microscopy showed the presence of multiple flagella in wild type cells (Fig 3C), while bactofilin mutant cells were devoid of any stained filaments (Fig 3D). Possibly, the synthesis of flagellin is disturbed in the bactofilin mutant cells. To test this idea, we performed Western blot analysis using anti flagellin antiserum. Fig 2C shows that flagellin levels are strongly reduced in bactofilin mutant cells.

In a next step, we were interested whether other components of the flagellum would also be missing in the bactofilin mutant strain. Please note that during exponential growth, a majority of PY79 cells (and bactofilin mutant cells) grow in a σ^D^ “off” state and form long chains, while only few cells shows motility [31]. We therefore imaged cells during late exponential growth, where most cells turn on the synthesis of flagellin and grow as short chains or single cells. A prerequisite for the filament assembly is the correct assembly of the flagellar hook that consists of the FlgE protein [25]. Therefore, we employed a strain containing a *flgE* allele that encodes FlgE with an engineered surface cysteine stainable by a fluorescent dye added to the cells [25]. In contrast to wild type cells, which showed a characteristic multi-spot staining along the membrane (Fig 3E), bactofilin mutant cells did not show any fluorescent dots (Fig 3G), but only homogeneous membrane staining, similar to wild type cells lacking the cysteine variant (Fig 3F), revealing that also the hook structures fail to be assembled in the absence of bactofilins. Therefore, one reason for the absence of the filament caused by the absence of bactofilins is the failure to establish a hook structure, because the absence of hook assembly affects the transcription of flagellin [32, 33].

We wondered if the loss of export of FlgE could be due to a failure to export flagellin, or in other words, if a possible effect of the absence of bactofilins on flagellin export may have an indirect effect on hook assembly. This concept has not been described for any other flagellar system, but we found it worth to investigate this formal possibility. To analyze this idea, we deleted the *hag* gene and investigated if hooks are still assembled. In a Δ*hag* background, clear FlgE foci could be detected (Fig 3H), showing that the absence of bactofilins affects the export of the hook protein independently from any putative effect on flagellin.

Finally, we tested whether assembly of the flagellar basal body would be affected by the lack of bactofilins. We used a FliM-CFP fusion expressed from the amylase locus (under control of the original *P*_*fla-che*_ promoter), previously reported to be functional [26] (Fig 1N), and generated YFP-FliG and YFP-FliY fusions that are integrated into the original gene locus and are therefore expressed as sole source of the proteins. The YFP-FliG fusion functionally complemented the wild type copy (Fig 1O), while the FliY fusion was not functional (Fig 1P). All three fusions showed a very similar pattern of localization (Fig 3I, 3J and 3K), therefore, the YFP-FliY fusion was also investigated as a marker for basal bodies. Fluorescent protein fusions were moved into the bactofilin mutant background, and cells were investigated by fluorescence microscopy. For all three fusions, we observed a fluorescence pattern similar to that of wild type cells (Fig 3L, 3M and 3N). In all cells, multiple foci were present at the lateral cell membranes, and less frequently at the cell poles. These experiments show that basal bodies assemble in a wild type fashion in the absence of bactofilins. In bactofilin mutant cells, fluorescent foci for FliM-CFP (Fig 3M) and for YFP-FliY (Fig 3N) were less clearly to be seen than in wild type cells, due to higher background fluorescence. However, the number of foci (between 7 and 15) was the same between mutant and wild type cells, and puncta intensities were approximately equal (wt: 403 ± 45 AU and mutant: 458 ± 15 AU), so the observed higher background fluorescence did not impact the quantity of basal bodies. These experiments indicate that bactofilins do not play a major role in the assembly of basal bodies. Thus, we show that bactofilins are essential for the formation of flagellar hook and filament, but not for the recruitment of basal body components.

### BacE and BacF differentially localize to defined sub 100 nm assemblies *in vivo*

**Fig 4:**
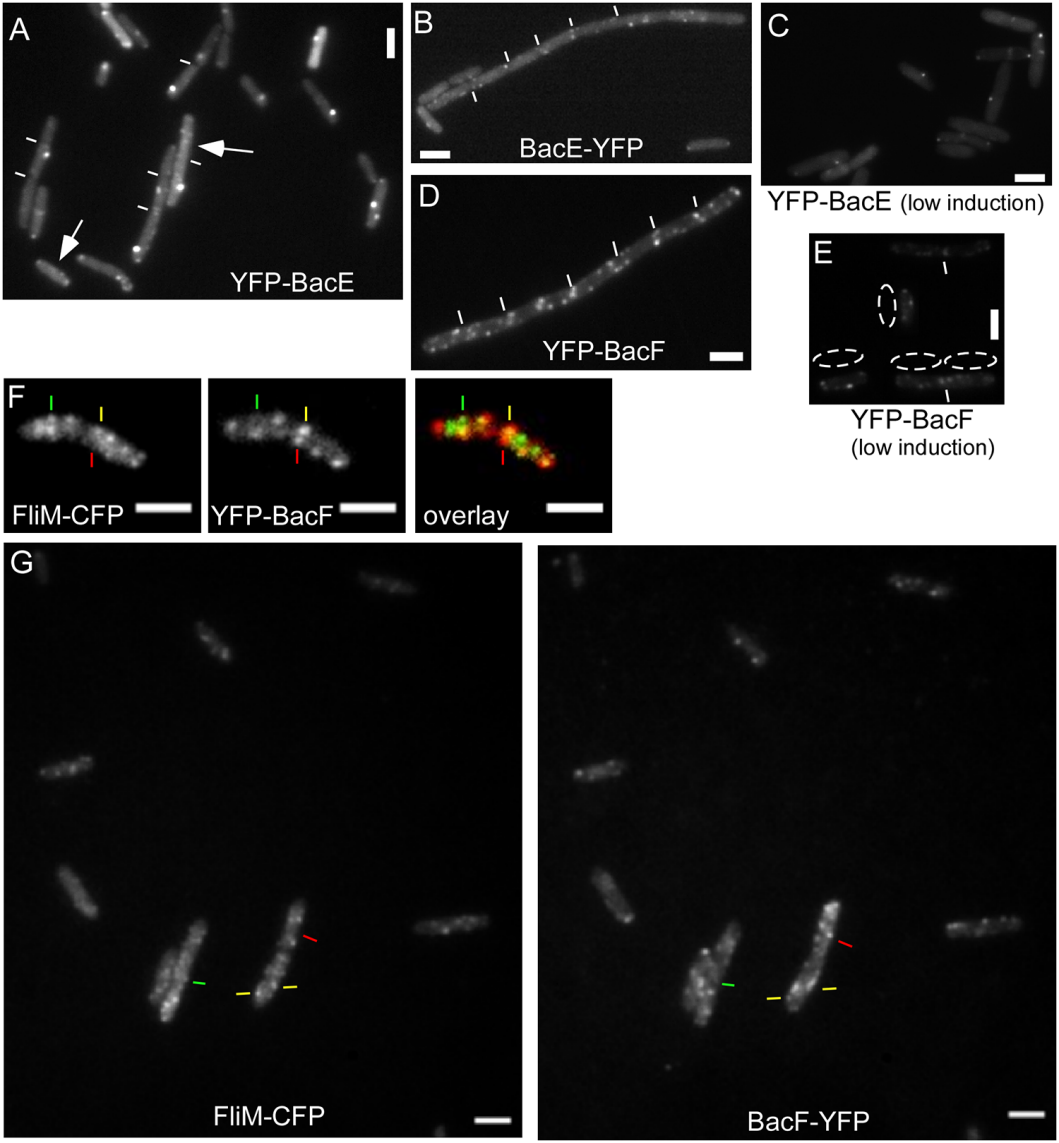
Localization of bactofilins in *Bacillus subtilis* cells during late exponential growth, expressing. A) YFP-BacE (white lines indicate septa between cells), arrows indicate the presence of several additional foci of low fluorescence intensity, which can be observed in about 20% of the cells (JEA102), B) BacE-YFP (JEA101), C) YFP-BacE under very low induction (JEA102), D) YFP-BacF (JEA104), E) YFP-BacF under very low induction (JEA104), dotted areas indicate cells next to the regions, F) coexpression of FliM-CFP (green in overlay) and YFP-BacF (red in overlay), green lines localization of FliM-CFP with no YFP-BacF signal, red lines localization of YFP-BacF with no FliM-CFP signal, yellow lines colocalization of both signals (JEA109). G) Field of *B*. *subtilis* cells expressing FliM-CFP and YFP-BacF (JEA109), line color as in panel F). White bars 2 μm.

Bactofilins from *C*. *crescentus* and *M*. *xanthus* have been reported to form membrane-associated assemblies *in vivo* and filaments *in vitro* [1, 4]. We performed fluorescence microscopy in live *B*. *subtilis* cells to study the pattern of localization of BacE and of BacF using the functional N-terminal fusions. We used 0.1% xylose as inducer, because this concentration resulted in an expression that was close to that of the C-terminal YFP fusions, which are under control of the original promoter (Fig 2B: band intensity BacE-YFP = 100%, YFP-BacE = 109.5%, BacF-YFP = 100%, YFP-BacF = 102.2%). YFP-BacE formed discrete foci in the cells, all of which localized to the border of the cells, suggestive of membrane association (Fig 4A). Besides a single bright focus, many cells contained several much weaker foci, but also detectable homogeneous fluorescence within the cytosol (Fig 4A). Interestingly, the semi-functional BacE-YFP fusion localized in a similar manner as the N-terminal fusion (Fig 4B). Western blot analysis showed that there is no free YFP present in the cells, only full length YFP-BacE, or full length YFP-BacF (Fig 2A).

In contrast to YFP-BacE, YFP-BacF formed several discrete membrane-proximal foci, but did not show visible fluorescence within the cytosol (Fig 4D), suggesting that most BacF molecules are membrane-associated. Thus, BacF localizes in a manner reminiscent of flagellar proteins, but the two bactofilins have somewhat dissimilar localization patterns. To rule out overexpression artifacts, we expressed YFP-BacE and YFP-BacF with a very low concentration of inducer (0.01% xylose), such that the signals were barely detectable. This way, YFP-BacE is expressed in a lower level than the semi-functional BacE-YFP fusion (which is driven by the original promoter), as verified by Western blotting (Fig 2A). Even low-level expression of both bactofilins resulted in a pattern of localization that was similar to that under full induction (Fig 4C for YFP-BacE, compare with Fig 4A, and Fig 4E for YFP-BacF, compare with Fig 4D), supporting the view that the localization of the N-terminal fusions represents the true subcellular pattern of bactofilins.

**Fig 5:**
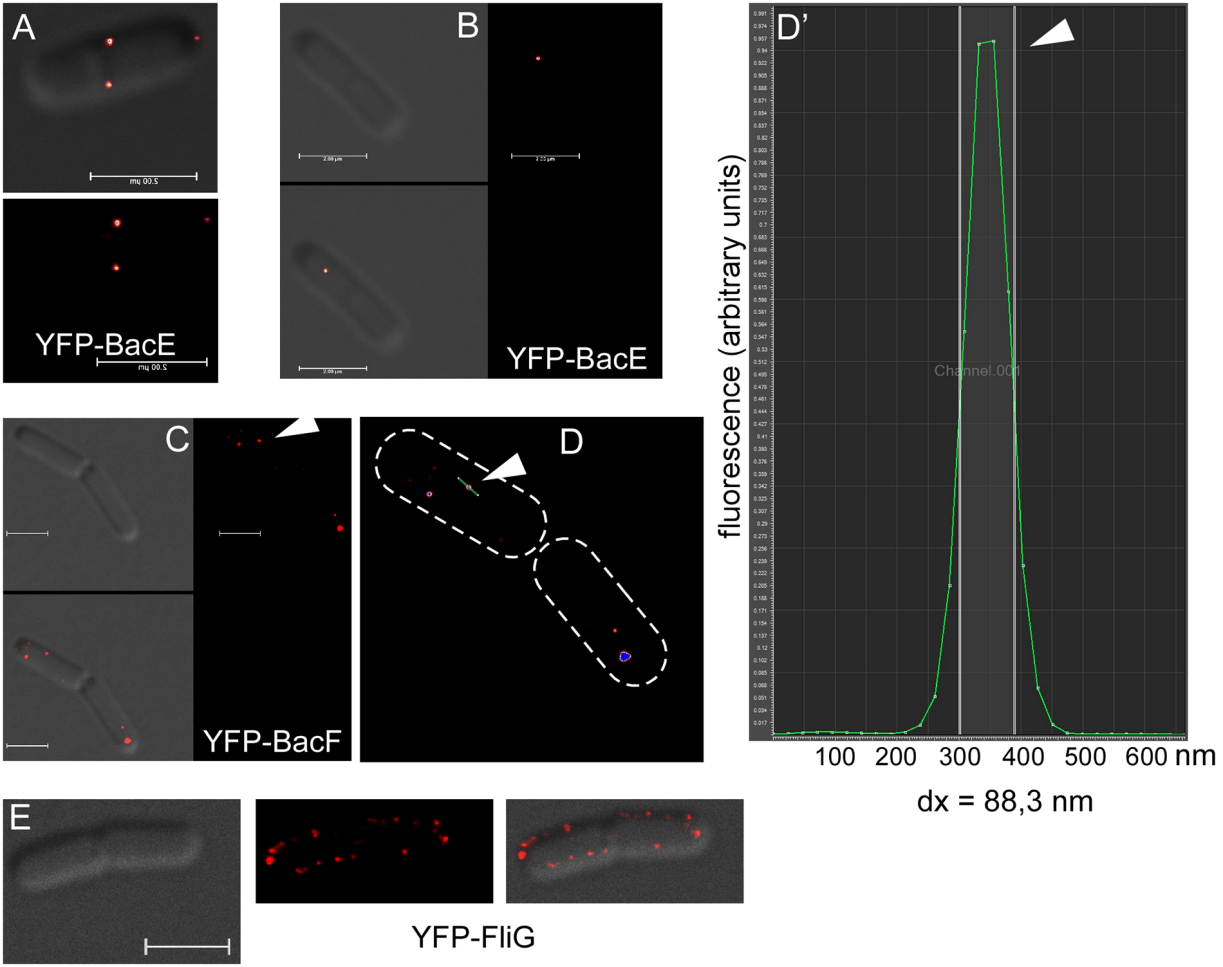
G-STED images in cells during late exponential growth A) and B) YFP-BacE (JEA102). A) upper panel overlay of bright field and fluorescence, lower panel fluorrscence, B) left upper panel bright field, right panel fluorescence, lower panel overlay, note that the focus is on the surface of the cell, C) YFP-BacF (JEA104), triangle indicates focus that is measured in panel D, D) YFP-BacF fluorescence from panel C, cell outlines are indicated by dashed lines, triangle indicated line through the focus, whose intensity is shown in panel D’, D’) intensity histogram relative to cell length, green line fluorescence intensity in arbitrary units, half maximal fluorescence is indicated by white vertical lines, whose distance (dx) is the size of the focus, in this case 88.3 nm. E) YFP-FliG (JEA105), left panel bright field, middle panel fluorescence and right panel overlay. White bars 2 μm.

Due to the limit of resolution of conventional fluorescence microscopy, fluorescent signals detected for YFP-bactofilin fusions could represent up to 250 nm long filaments, which might confer e.g. an anchor-like function for the assembly of the flagellum. Therefore, we employed G-STED (gated stimulated emission depletion) microscopy, which allows visualization of YFP signals down to 42 nm [34]. In contrast to MreB, which forms clearly visible filaments using STED [34], YFP-BacE and YFP-BacF signals appeared round rather than elongated, which argues against the formation of filamentous structures (Fig 5A, 5B and 5C). The size of fluorescent structures can be determined from the size of a fluorescent signal at half of the height of the fluorescence peak, which comprised >90% of the fluorescent molecules (Fig 5D and 5D’). From 60 signals, we determined an average size of 78 nm, with bactofilin assemblies ranging from 70 nm to (rarely) 130 nm. Thus, both bactofilins form defined assemblies of a large size; for comparison, a bacterial ribosome is about 20 nm large [35]. When YFP-BacE was induced as an additional copy in the *B*. *subtilis* cell, some cells showed large filamentous structures, which were mobile within the cells (S1 Movie), showing that upon overproduction, *Bacillus* bactofilins can also form filamentous structures.

One to five signals for YFP-BacE per cell were detectable by G-STED, while generally, more signals for YFP-BacF (between 3 and 12) were present on the cell surface (Fig 5A, 5B and 5C). Representing the flagellar basal body itself, YFP-FliG was measured to have a size of around 90 nm (50 foci analyzed). Interestingly, about 20 YFP-FliG foci per cell were observed at the focal plane (Fig 5E, 45 cells analyzed). Thus, there are more visible basal body structures in *B*. *subtilis* cells than bactofilin assemblies.

### BacE and BacF dynamically colocalize with the flagellar basal body

**Fig 6:**
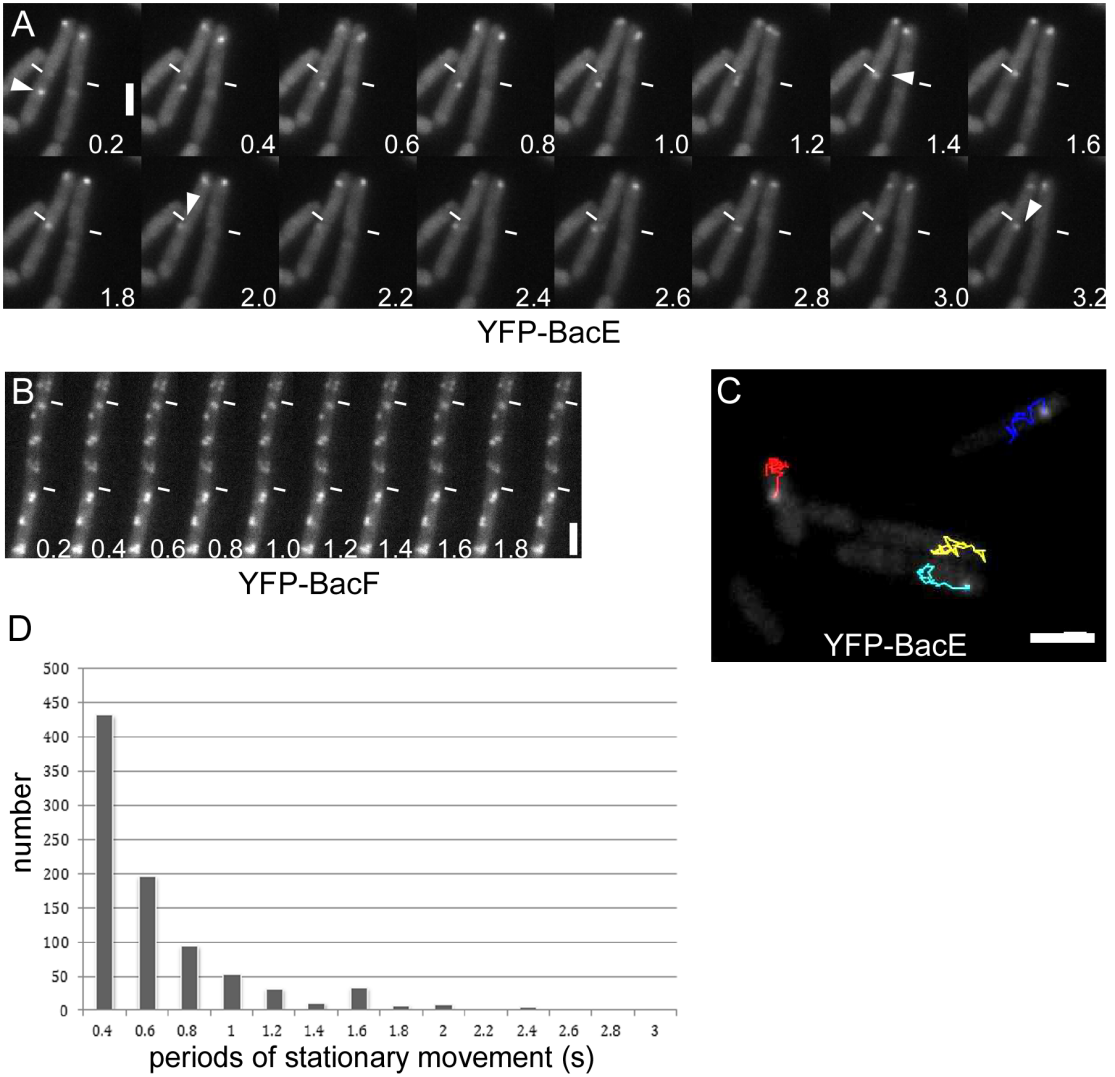
Dynamics of bactofilins in cells during late exponential growth. A) and B) montages of 200 ms stream acquisitions of A) YFP-BacE (JEA102) and B) YFP-BacF (JEA104). Numbers indicate time in seconds. The white triangle indicates movement of a YFP-BacE signal. C) Tracking of YFP-BacE foci. Lines of different colors show tracks of individual signals (48 frames of a stream of 0.2 s intervals). D) Bar diagram shows the number of tracking points of a YFP-BacE molecule that arrest for a defined time interval as stated on the x-axis.

Our data indicated a putative role of the bactofilins in flagellar assembly, in particular for the hook and filament structures. To obtain further insight into this process, we performed high-resolution fluorescence microscopy to investigate the cellular relationship of the bactofilins with the flagellar basal body that contains the fT3SS. We employed dual labeling of YFP-BacF with the functional FliM-CFP fusion as a marker for basal bodies/fT3SS. Interestingly, only 35% of the YFP-BacF signals colocalized with FliM-CFP (in 250 cells analyzed), the remaining signals were clearly distinct (Fig 4F and 4G). Images were taken with negligible time delay, switching between 445 nm and 515 nm lasers and using a single dual color filter cube. Moreover, less than 20% of FliM-CFP or of YFP-BacF foci showed considerable movement in the frame of seconds: in fast time lapse experiments, YFP-BacF assemblies were largely static in between 0.2 second intervals (Fig 6B and S2 Movie). This was also the case for YFP-FliG (S3 Movie) and for FliM-CFP, for which only two to three frames could be acquired due to rapid bleaching. Because FliG and FliM are part of the same structure, we are inferring that the movement of FliG is characteristic of basal bodies. Interestingly, YFP-BacE was highly mobile, in that usually, a single visible focus moved between different parts of the membrane between 0.2 second intervals (Fig 6A, S4 and S5 Movies). A frequently observed pattern was the localization of YFP-BacE to the cell center for several seconds, and diffusion to the lateral cell membrane (S4 and S5 Movies), or movement to and from the cell pole and changes between the lateral sides of the cells (Fig 6A). These data show that BacE and BacF exhibit a strongly divergent dynamical behavior along the cytoplasmic face of the cell membrane. The colocalization of BacE with the basal body is likely transient, whereas BacF shows a rather static interaction, however, with only a subset of basal bodies.

Unfortunately, it was not possible to obtain satisfactory time lapse acquisitions with FliM-CFP, as fluorescence bleached rapidly, such that during the second exposure, most foci could no longer be clearly seen. However, because most YFP-FliG foci were static (and thus likely also FliM-CFP), we imaged a strain expressing FliM-CFP and YFP-BacE simultaneously, acquiring one CFP frame and ensuing 200 ms intervals of YFP fluorescence. S6 and S7 Movies show that YFP-BacE foci moved in between FliM-CFP foci, and arrested at the latter positions for few hundred milliseconds only. Given the caveat that some FliM signals may have moved (but possible to another position where BacE can be seen to arrest), it is still reasonable to state that BacE can interact with basal bodies—as determined by colocalization—in a sub-seconds range. In order to obtain more quantitative data, we tracked YFP-BacE molecules using ImageJ software (Fig 6C and S8 Movie), which documents intervals of extensive movement or of arrest. We scored the number of YFP-BacE signals that arrested for several time points (Fig 6D), which illustrates that few assemblies are statically positioned for more than a second, while a vast majority arrests for 400 to 800 ms at a particular place, including a basal body.

### BacE and BacF can form filamentous structures in a heterologous cell system

**Fig 7:**
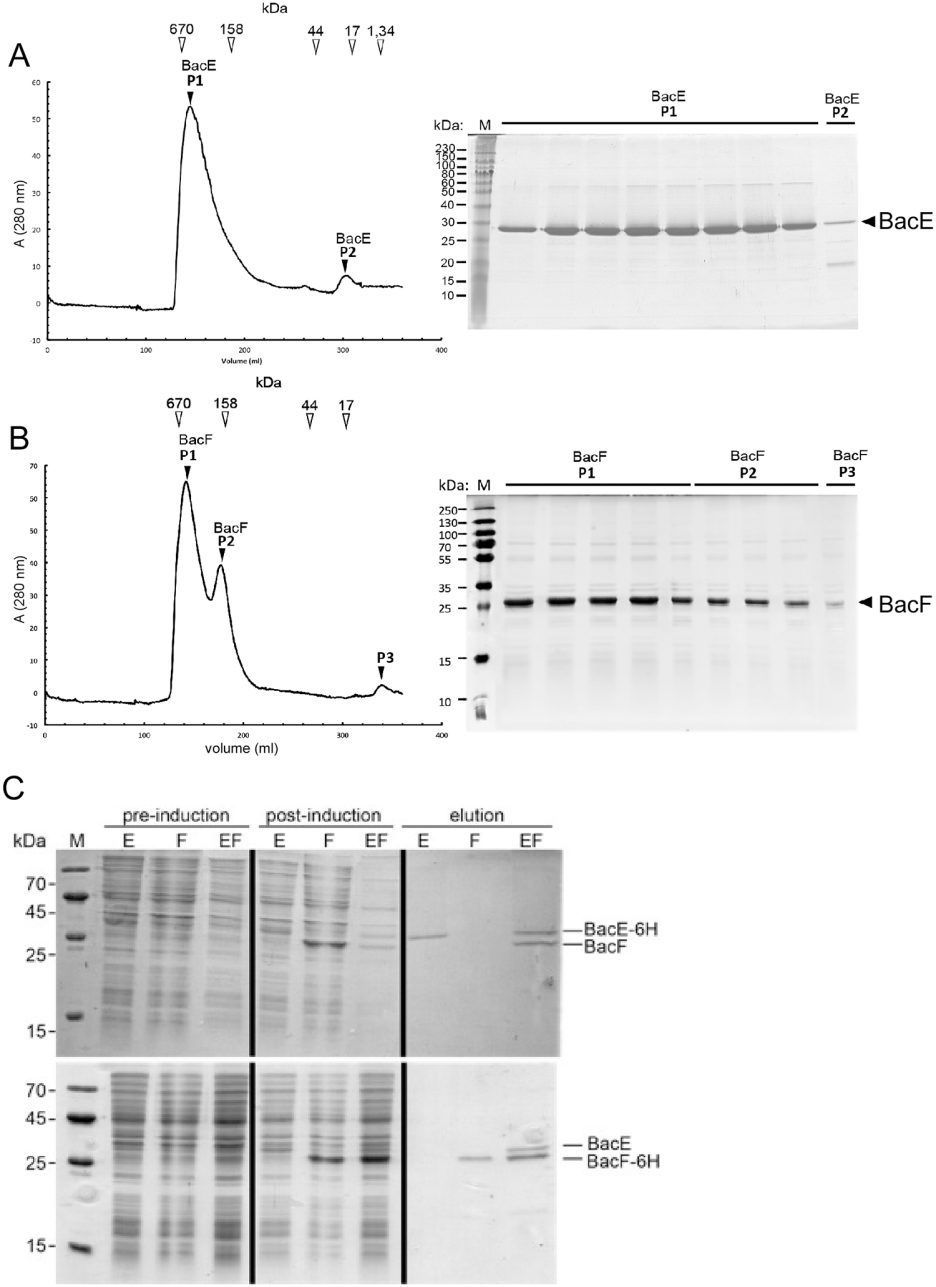
Biochemical analysis of bactofilins. A) Size exclusion chromatography (SEC) of BacE. Elution volumes of marker proteins are shown above the spectrum, elution fractions are shown in the corresponding Coomassie-stained SDS-PAGE. Migration of BacE or of BacF is indicated on the right of the gels. B) SEC analysis of BacF, see panel A for description. C) Co-purification of BacE/BacF complex by Nickel-NTA columns. E refers to BacE, F to BacF, EF to coexpression of both proteins; upper panel: BacE carries a hexa-histidine tag, while BacF is not tagged, lower panels: BacF carries a hexa-histidine tag, while BacE is not modified. Pre/Post-induction: cell extract from *E*. *coli* cells before or after induction of BacE, BacF or of both proteins, elution shows that the hexa-histidine fusions bind to the column, or both proteins when only one carries the affinity tag, but not the non-fusion protein by itself. Migration of BacE or of BacF is indicated on the right of the gels.

To gain more insight into the biochemical properties of BacE and of BacF, both proteins were purified to apparent purity and subsequently were subjected to analytical gel filtration. Both proteins eluted from columns near and within the void volume (Fig 7A and 7B). Only a minor fraction of BacE or of BacF eluted as monomers (Fig 7A and 7B). Therefore, the proteins formed large assemblies or aggregates, indicating that the proteins may build up extended filamentous structures, as described for other members of this newly recognized protein family [2], or may aggregate when present on their own.

**Fig 8:**
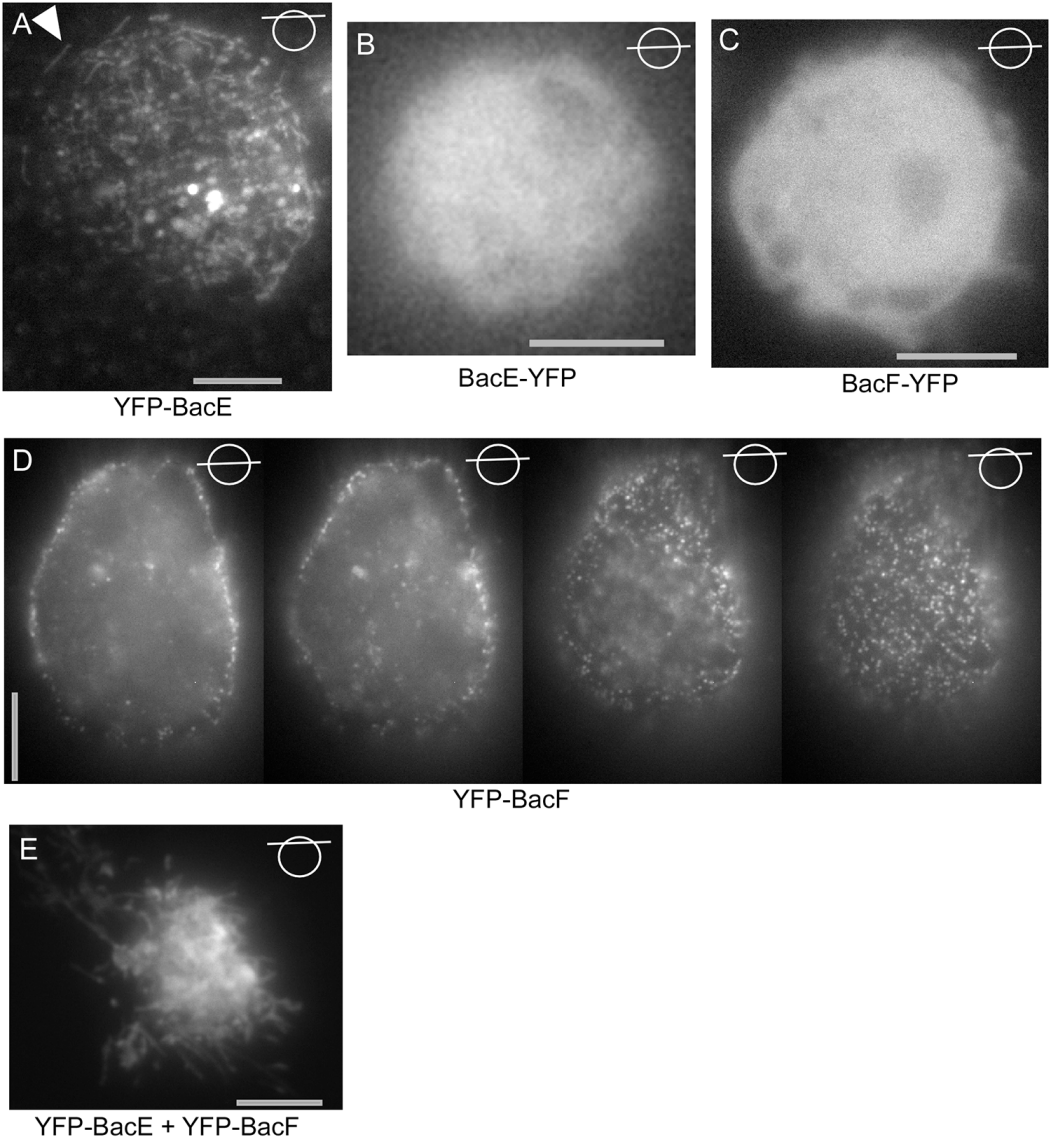
Heterologous expression of YFP-BacE and YFP-BacF in S2 Schneider cells. Lines through circles indicate the focal plane of the image. A) YFP-BacE, filamentous structures are indicated by a triangle, B) BacE-YFP, C) BacF-YFP, D) partial Z-stack through an S2 cell expressing YFP-BacF. E) Cells co-expressing YFP-BacE and YFP-BacF. Grey bars 5 μm.

We next used a heterologous expression system (S2 Schneider cells) derived from Drosophila macrophages [28], to investigate the localization pattern of bactofilins in cells devoid of any potential cofactor from a bacterial cell. Interestingly, YFP-BacE showed an intrinsic affinity to the cell membrane, where it occasionally assembled into filamentous structures (Fig 8A), in contrast to BacE-YFP (Fig 8B), which showed diffuse localization; this fusion is only partially functional in *B*. *subtilis* cells (Fig 1I). YFP-BacF also assembled into foci at the cell membrane (Fig 8D), and therefore also has intrinsic membrane affinity, like BacE. The non-functional BacF-YFP fusion was diffusely distributed within the cytoplasm (Fig 8C), showing that membrane-association is not a YFP artifact. However, YFP-BacF did not form any filaments in S2 cells, like YFP-BacE. Interestingly, when co-expressed, extensive filamentous structures were observed (Fig 8E and S9 Movie), indicating that a) BacE and BacF may interact with each other and b) they can form extended filaments in a heterologous cell system. To further prove a direct interaction between the two bactofilins, the proteins were co-expressed in *E*. *coli* cells, with BacE carrying a hexa-histidine tag, while BacF did not have any affinity tag, and *vice versa*. Fig 7C shows that both proteins eluted from Ni-NTA column chromatography in a stoichiometric manner (upper panel, lane “elution EF”), while BacF did not bind to the column by itself (Fig 7C, lane “elution F”). In the reciprocal experiments (Fig 7C, lower panels), BacE did not bind to the column (lane “elution E”), but eluted together with 6-His-BacF (lane “elution EF”). These results show that BacE and BacF interact with each other *in vitro*, and are able to form filamentous structures when expressed at high levels, although there is no evidence for filament formation in *B*. *subtilis* cells at physiological expression levels (Fig 5A–5C).

## Discussion

Our work provides new conceptual insight into the function of bactofilins, a protein family with widespread members, found in most bacterial phyla. In spite of their wide conservation, and their importance in cell shape maintenance and motility, only little is known about how they operate mechanistically. Here, we show that both the *Bacillus subtilis* bactofilins, BacE and BacF, confer an essential function in the assembly of the flagellar hook- and filament structures, explaining their importance in motility of *B*. *subtilis*. While at present, we cannot explain how bactofilins may affect the export or assembly of the hook structure, a failure to set up this part of the flagellum will also prevent the formation of the filament [11, 14].

Both proteins seem to execute their important function(s) at the flagellar basal body, because they both show partial colocalization with proteins from this structure. However, basal bodies seem to be correctly assembled, based on the recruitment of the three components we imaged, even in the absence of both bactofilins. These experiments indicate that bactofilins operate more directly at the level of export of FlgE (component of the hook) and of flagellin. Because flagellin cannot be exported in the absence of a completed hook structure, bactofilins may exclusively act during FlgE export, and may have an indirect role on the secretion of flagellin.

A further important finding of this work is the fact that *B*. *subtilis* bactofilins can form extended filaments when expressed at high level in a heterologous cell system that lacks any bacterial protein cofactors. Co-expression of BacE and of BacF increased the level of filament formation seen for BacE alone, indicating a productive interaction between the two paralogs. Indeed, we show that BacE and BacF directly bind to each other *in vitro*. This was also demonstrated for BacA and BacB found in *C*. *crescentus*, which copolymerize and interact *in vitro* [36]. However, super resolution fluorescence microscopy in *B*. *subtilis* cells, where bactofilin YFP fusions were expressed at physiological levels, revealed that bactofilins are part of 60 to 70 nm large assemblies, of which they may be the central component, based on their ability to multimerize *in vitro*. Therefore, bactofilins do not form extended or elongated filaments in *Bacillus* cells, but defined assemblies.

BacE forms fewer subcellular assemblies than BacF, which is even outnumbered by the existing basal bodies/flagella. Thus, bactofilins are not integral components of the flagellum, but likely transiently interact with basal bodies in order to confer their essential role in filament assembly, for which both proteins are required.

A major finding of our work is the dynamic localization of BacE, which moves between different sites along the lateral cell membrane and the cell pole, resting at distinct positions for no longer than few hundred milliseconds at a time. Therefore, BacE is the first member of bactofilins that shows high mobility within a cell. By contrast, BacF forms more static membrane-associated assemblies, although a subfraction of these is also mobile underneath the membrane. Based on the partially co-localization of BacF with basal bodies, and based on the dynamics of BacE, we speculate that the proteins may confer a switch-like process at the type III flagellar secretion system, to set this system ready for export of the hook. Alternatively, bactofilins may provide an assembly platform for the secretion apparatus, in agreement with their propensity to multimerize *in vitro*. If this is the case, this platform must operate extremely efficiently, because BacE only arrests at distinct sites including basal bodies for few hundred milliseconds.

Taken together, our work defines a conceptually new role of bactofilins in regulating steps of flagellar export in *B*. *subtilis*. The finding that bactofilins form assemblies of 70 nm size and show a mobile localization pattern, especially in case of BacE, suggest that in addition to an assumed scaffolding function observed for other bacteria [1, 3, 4], bactofilins can drive reactions and/or assemblies in a highly dynamic manner, possibly in many other bacterial species.

## Supporting Information

S1 Movie YFP-BacE overexpressed in *B*. *subtilis* cells. (AVI)

S2 Movie YFP-BacF expressed in *B*. *subtilis* cells. (AVI)

S3 Movie YFP-FliG expressed in *B*. *subtilis* cells. (AVI)

S4 Movie YFP-BacE expressed in *B*. *subtilis* cells. (AVI)

S5 Movie second example for YFP-BacE expressed in *B*. *subtilis* cells. (AVI)

S6 Movie *B*. *subtilis* cells expressing YFP-BacE and FliM-CFP. (MOV)

S7 Movie Cells expressing YFP-BacE and FliM-CFP. (AVI)

S8 Movie Tracking of YFP-BacE. (AVI)

S9 Movie Z-stack through an S2 cell expressing BacE-YFP and BacF-YFP. (AVI)

S1 Table *E*. *coli* strains and plasmids used in this study. (DOCX)

S2 Table Primers used in this study. (DOCX)
