# Supplementary material for: Bacillus subtilis Bactofilins Are Essential for Flagellar Hook- and Filament Assembly and Dynamically Localize into Structures of Less than 100 nm Diameter underneath the Cell Membrane
Source: PLoS One. 2015 Oct 30;10(10):e0141546. doi: 10.1371/journal.pone.0141546 (PMC4627819; doi:10.1371/journal.pone.0141546)
Supplement: S1 Table — (DOCX) [file pone.0141546.s010.docx]

**S1 Table. *E. coli* strains and plasmids used in this study.**

Strain Source reference

| ***E. coli* strains** | |
| --- | --- |
| XL1-Blue | Stratagene |
| BL21 Star (DE3) | Invitrogen |
| **Plasmids** | |
| pSG1164 | [[1](#_ENREF_1)] |
| pSG1729  pSG1193  pSG30 | [[1](#_ENREF_1)]  [[2](#_ENREF_2)]  [[3](#_ENREF_3)] |
| pET16b  pGAT3 | Novagen  Novagen |
| pDG1515  pFD1  pOTJ1 | [[4](#_ENREF_4)]  [[5](#_ENREF_5)]  this study |

**References**

1. Lewis PJ, Marston AL (1999) GFP vectors for controlled expression and dual labelling of protein fusions in *Bacillus subtilis*. Gene 227: 101-110.

2. Feucht A, Lewis PJ (2001) Improved plasmid vectors for the production of multiple fluorescent protein fusions in *Bacillus subtilis*. Gene 264: 289-297.

3. Guttenplan SB, Shaw S, Kearns DB (2013) The cell biology of peritrichous flagella in *Bacillus subtilis*. Mol Microbiol 87: 211-229.

4. Guerout-Fleury AM, Shazand K, Frandsen N, Stragier P (1995) Antibiotic-resistance cassettes for *Bacillus subtilis*. Gene 167: 335-336.

5. Dempwolff F, Reimold C, Reth M, Graumann PL (2011) *Bacillus subtilis* MreB orthologs self-organize into filamentous structures underneath the cell membrane in a heterologous cell system. PLoS One 6: e27035.
