# Supplementary material for: Bacillus subtilis Bactofilins Are Essential for Flagellar Hook- and Filament Assembly and Dynamically Localize into Structures of Less than 100 nm Diameter underneath the Cell Membrane
Source: PLoS One. 2015 Oct 30;10(10):e0141546. doi: 10.1371/journal.pone.0141546 (PMC4627819; doi:10.1371/journal.pone.0141546)
Supplement: S2 Table — (DOCX) [file pone.0141546.s011.docx]

**S2 Table. Primers used in this study**

| Primer | Sequence 5’-3’ |
| --- | --- |
| 2213 | ACTGGGCCCGGTTCAGGAAAGATACATGG |
| 2214 | ACTGAATTCCAACTTTGTGGATGTTTCAAC |
| 2215 | ATGGGGCCCATGGATGTAGTGGAAAAGCT |
| 2216 | ATGGAATTCCAGCATTCCTTTTCTGCAG |
| 2238 | ACTGGGCCCATGGAGACAACAAAACTGGG |
| 2239 | ACTGAATTCAAAACTCGCGCGTCTTTTCA |
| 2240 | ACTGGGCCCGAATGTAAAGGCGATCTGACA |
| 2241 | ACTGAATTCCAGTTTTGTTTTTTCTTTAATCTG |
| 2258 | ACTGGGCCCTTTATTACAAAATTTGGCCAG |
| 2259 | ACTGAATTCCGGCAATATCCTGAGATTT |
| 2573 | ACTGATATCCAACTTTGTGGATGTTTCAAC |
| 2574 | ACTGATATCCAGTTTTGTTTTTTCTTTAATCTG |
| 2926 | ACTGGGCCCATGGCGAGACGTGATCAA |
| 2927 | ACTGAATTCCGTTCTATCCATGACCGC |
| 4168 | ACTAAGCTTCTGTTATAAAAAAGGATCAATTT |
| 4169 | ACTAAGCTTCTCTAGAACTAGTGGATCC |
| 4305 | ACTGGATCCTTACAACTTTGTGGATGTTTC |
| 4306 | TTAACCATGGGCCACCATCACCATCACCATATGGAGACAACAAAACTGGG |
| 4307 | ACTCCATGGATGGAGACAACAAAACTGGG |
| 4308 | ACTGGATCCTTACAGTTTTGTTTTTTCTTTAA |
| 4309  4310 | TTAACCATGGGCCACCATCACCATCACCATATGGATGTAGTGGAAAAGCT  ACTCCATGGATGGATGTAGTGGAAAAGCT |
| 4313 | ACTGGGCCCATGGCGATCCAAAATCCATATACAGCCT |
| 4314 | ACTGAATTCTGCGATCCCGCCTGATC |
| 4341 | ACTGAATTCTTACAGTTTTGTTTTTTCTTTAATC |
| 4410 | ACTCTCGAGTTACAACTTTGTGGATGTTTC |
| 4411 | ACTCTCGAGTTACAGTTTTGTTTTTTCTTTAA |
| 4783 | ACTGGGCCCATGATCATTCATACGAAGTAC |
| 4784 | ACTGAATTCGCATGATTCTCCTCCAATC |
| 4922 | ACTGGGCCCATGGAGAATAATAGATTATCTC |
| 4923 | ACTGAATTCTGTACCCTTTTCTTCAGTAA |
| 4968 | ACTGGGCCCATTGAGGATATGGGTGCTG |
| 4997  5203 | ACTCTCGAGACGTAATAATTGAAGTACGTTT  ACTGAATTCTTACAACTTTGTGGATGTTTC |
